# Experience from the First Live-Birth Derived From Oocyte Nuclear Transfer as a Treatment Strategy for Mitochondrial Diseases

**DOI:** 10.4172/1747-0862.1000258

**Published:** 2017-05-02

**Authors:** J Slone, J Zhang, T Huang

**Affiliations:** 1 Division of Human Genetics, Cincinnati Children’s Hospital Medical Center, 3333 Burnet Avenue, Cincinnati, Ohio-45229, USA; 2 New Hope Fertility Center, 4 Columbus Circle, New York, NY10019, USA

## Introduction

Our latest breakthrough involves the successful application of mitochondrial replacement therapy (MRT) and has attracted worldwide attention [1]. This has also raised a considerable debate regarding the safety of mitochondrial replacement therapy. In particular, there is a concern about carryover of small amounts of the mother’s mutant mtDNA into the baby, and whether the levels of this mutant mtDNA can drift over time to replace the donated, healthy mtDNA (thus defeating the entire purpose of the procedure). Such heteroplasmy drift could occur in a variety of ways; however, the most commonly debated mechanism involves nuclear-mitochondrial incompatibility. That is, in cases where nuclear transfer occurs between oocytes of women from widely divergent haplogroups, there is a possibility that the newly created combination of nuclear and mitochondrial genomes may experience deleterious interactions. Whether this originates from the mother’s nuclear genome or the donor’s mitochondrial genome, the ultimate effect would be the same; a selective pressure that favors the proliferation of the original, mutant mtDNA. It has been reported in several recent *in vitro* studies that, even though the low levels of heteroplasmy introduced into human oocytes often vanish, they can sometimes result in mtDNA genotypic drift and reversion to the original genotype in some of the reconstituted human embryonic-derived stem cell (hESC) lines [2–5]. There is, however, a serious question of the rigor of these studies and whether these *in vitro* experiments reflect the *in vivo* applications.

## How Important is the Carryover Rate in MRT?

**Figure 1:**
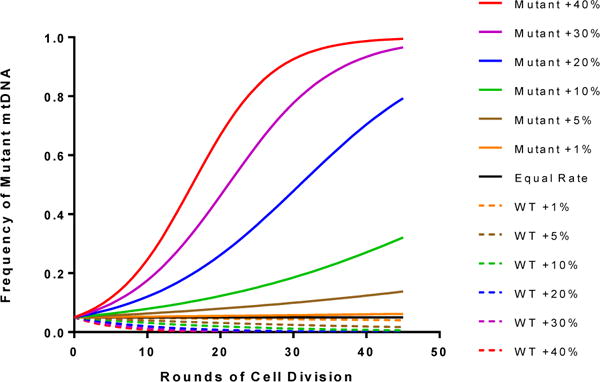
Moderate differences in mtDNA proliferation rate can lead to massive shifts in heteroplasmy during human development.

Several groups have emphasized the importance of keeping a low carryover rate to obtain the best clinical outcome. However, the mtDNA heteroplasmy shift trend could be much more important to determine than the initial carryover of mutant mtDNA observed in the reconstituted embryos. An adult human has a total number of 3.72 × 10^13^ cells [6], harboring 10^17^ mitochondria [7]. To produce this number of cells, approximately 45 rounds of cell division would be required at a minimum. Furthermore, given that each cell contains hundreds or thousands of copies of mtDNA that must be replicated, the mitochondrial genome likely undergoes several rounds of replication during each round of cell division (and, in fact, continues to replicate even in terminal differentiated cells), dramatically inflating the number of replication events experienced by each copy of mtDNA. This is without accounting for the large number of additional divisions required to replace cells destroyed by apoptosis or cell death (especially in highly proliferative tissues), or the additional mtDNA replications required to replace mtDNA lost when mitochondria are destroyed by mitophagy and other mechanisms, which will further expand these numbers. Given these very conservative parameters, simple mathematical modeling easily demonstrates that a slight selective advantage for a low abundance mtDNA can rapidly lead to an increase in the final level of heteroplasmy in an adult (Figure 1). Such advantages can be the result of more rapid mtDNA replication rates, increased cell survival, or increased cell proliferation for a given mtDNA [4]. Importantly, the biological basis underlying the occasional mutation load drift event still remains to be established and likely differs between specific mitochondrial mutations.

## Conditions to Consider for MRT: Leigh Syndrome (T8993G) vs (A3243G)

Heteroplasmic alleles can shift during mitotic and meiotic cell divisions, a process known as replicative segregation. To date, the segregation of mtDNA heteroplasmy is the most unpredictable aspect of mitochondrial genetics. However, previous evidence-based studies provided basic understanding of the segregation pattern of several common mtDNA pathogenic mutations. Previous studies have recognized the variability of pathogenic mtDNA: that is, mutations behave and are segregated differently in somatic tissues and preimplantation embryos [8,9]. In fact, the most severe somatic mtDNA mutations may be selectively eliminated during oogenesis, preventing the most severe mtDNA mutations from being passed on across generations while permitting more moderate (but still significant) mutations to be passed on from mother to child [10]. Thus, it is imperative that the medical community factor the specific biological context of the preimplantation embryo into any discussion of the genetic risks of mitochondrial replacement therapy.

The studies initiated on the potential of pre-implantation genetic diagnosis for women who are heteroplasmic for the MELAS (A3243G) and the NARP/Leigh syndrome (T8993G) mutations have concluded that the percentage of heteroplasmy for these two mutations did not differ significantly among blastomeres of embryos, that various fetal tissues had similar heteroplasmy levels, and that heteroplasmy levels did not change with gestational age [11–14]. The mtDNA T8993G mutation, one of the most common mutations in Leigh syndrome, impairs mitochondrial function. In cells harboring a high percentage of heteroplamy for the T8993G mutation, mitochondrial ATP synthesis can reduce the Fo portion of ATPase activities by 50–70%, therefore resulting in failure of the mitochondrial respiratory chain and ATP-synthetic defects [15]. Patients with the T8993G mutation associated with Leigh syndrome often develop regression of both mental and motor skills, leading to disability and rapid progression to death, often due to seizures and respiratory failure [15]. However, when mtDNA T8993G mutation load is less than 30%, the carrier is expected to be asymptomatic, and severe symptoms do not occur until the heteroplasmy level reaches 60%–70% [16], suggesting a high tolerance threshold for mutation load.

An independent study confirmed that the heteroplasmy levels for T8993G mutation remains stable during pregnancy [17]. No substantial tissue variation was found, nor was there any substantial change in the proportion of this mutation over time (8–23 years) in four individuals [18,19]. In contrast to the ATP6 T8993G mutation, the potential for inter-tissue differences in heteroplasmy levels for the A3243G mutation may be significantly higher. The heteroplasmic mutant A3243G mtDNA progressively increased in frequency in six different cybrid lines [20]. In a case report, a woman harboring 35% of the A3243G mutation requested preimplantation genetic testing (PGD). One embryo with trophoblast heteroplasmy levels of 12% was chosen for the procedure. This resulted in a successful delivery of a child for which a buccal cell DNA analysis at 1 month revealed a low heteroplasmy level (15%) and remained low in the first year of life [12]. However, a subsequent patient follow-up revealed a significantly higher mutation load in the child’s blood and urine (42%~52%) [21]. Given the low threshold tolerance for A3243G mutation, which has been reported to be as low as 45% in the literature [22], such a pronounced shift in mutant allele frequency is cause for concern.

## An Implausible Result?

The article on mtDNA drift published in *Cell Stem Cell* made national headlines. In that study, human embryonic pluripotent stem cell lines derived from blastocysts with expanding trophectoderm and a distinct inner cell mass were used to determine the replicative stability of the mtDNA genotype. The authors found that in seven of the eight cell lines, the mitochondrial heteroplasmy decreased below the limit of detection by passage 6 and remained stable for more than 30 passages, or more than 6 months of culture. However, the eighth cell line exhibited truly remarkable behavior. For this particular cell line, the carryover was 1.3% at derivation and remained low until more than 20 passages, then suddenly expands to 53.2% at passage 36, but then, the level decreases to 1% at passage 59 without any specific reason [3]. We find this result to be highly implausible, in particular because the mechanism by which mtDNA in this particular cell lines expanded to such a high level of heteroplasmy and then reduced dramatically has never been fully explained. The authors proposed the hypothesis that specific mitochondrial-nuclear combinations confer cellular survival and/or proliferative advantages [3]. However, the conclusion was that the nucleus from the different haplotypes did not confer a survival or proliferation advantage. In our opinion, while this study has drawn tremendous attention to the field, the rigor of the study and its results in terms of its intra and inter-group reproducibility are in question, and this discovery should be treated with the utmost caution. Critically, we observe that this *in vitro* study describes an implausible mtDNA behavior *in vivo* where the cell-cell interaction is critical for embryo development and the cell proliferation rate is much lower.

## Inconsistency of the Haplogroup Match

Last year, the Huang group, in collaboration with the Mitalipov laboratory, examined the importance of haplogroup matching for nuclear–mitochondrial interaction [23]. During human migration, mtDNA has undergone a series of mutations as adaptations to the environment. Some changes are neutral polymorphic variants, while other mtDNA alterations affect mitochondrial function. The difference between distant human mtDNA haplogroups includes up to 95 SNPs in the most extreme case [24]. D4a is a descendant from the M macro-haplo-group, while the F1a comes from the N macro-haplo-group, per the human mtDNA mutation tree. We examined compatibility of the nucleus from the D4a haplotype and mitochondria from the F1a with 47 different SNPs in mtDNA between two haplogroups. We found two such distant nuclei and mtDNA have normal nuclear–mitochondrial interaction as demonstrated by lineage-specific differentiation and restoration of metabolic activity [23].

Subsequent to the aforementioned work, Hyslop et al. reported mtDNA drift in human embryonic stem (hES) cell lines derived from early pronuclear transfer blastocyst. They found one line (out of a total of five) from a blastocyst with a 4% mtDNA carryover with an upward drift to approximately 20% by passage 12. However, a wide variation in heteroplasmy levels between colonies was observed. Interestingly, the karyoplast and cytoplast donors for that particular line were from the same mtDNA haplogroup (karyoplast:cytoplast, H:H), supporting our previous results that the haplogroup match may not be sufficient to prevent mtDNA drift. Thus, there is a possibility that other previously undescribed non-haplogroup sequence variants in mtDNA conferred a replicative advantage.

The recent work also suggested that some sequence variants in mtDNA may be critical. ES cell lines were generated from two siblings from spindle transfer blastocysts. A combination of maternal U5a and donor H1b mtDNA from one sibling displayed high levels of maternal mtDNA. In contrast, another sibling’s ES cell line created from a maternal U5a and donor V3 maintained the donor mtDNA, suggesting that the haplogroup mismatch may be associated with mutation mtDNA drift [4]. Furthermore, we discovered that the deletion of a single guanosine residue (maternal G6AG8 vs. donor G5AG8) in the conserved sequence box II (CSBII) resulted in a fourfold reduction of the replication primer synthesis in donor mtDNA [4]. CSBII is a sequence located at positions 299–315 in the mitochondrial genome that plays a crucial role in mtDNA replication, specifically the interplay between mitochondrial transcription termination and generation of the replication primer required to initiate heavy strand synthesis [25]. The CSBII contain a sequence at positions 303–315 that is highly variable between individuals, even within a haplogroup. Although this conserved sequence box II (CSBII) sequence is in the mtDNA of individuals from certain haplogroups, but not the haplogroup per se, it provides a more efficient replication process and subsequently confers a replicative advantage. Therefore, it is imperative to match the CSBII between the donor and mother.

The phenomenon of mtDNA drift is complicated in that it involves more than just the haplogroup or the CSBII sequence. One ES cell line derived by somatic cell nuclear transfer displayed a gradual increase in maternal (somatic) mtDNA from 19% (passage 2) to 100% (passage 10) of the total mtDNA content in a few passages. Sequence analysis did not reveal any CSBII SNP differences in these reversed cell lines. However, we noticed that clones with higher maternal mtDNA levels exhibited significantly faster growth rates (P<0.05) than those with lower maternal mtDNA [4]. This suggested that certain mtDNAs confer ES cells with faster growth and proliferative advantage, but it is independent of the conserved sequence box II.

In the same study, one cell line from one sibling (U5a) demonstrated 20% maternal H49 mtDNA. This mtDNA gradually increased during extended culture to 90% (passage 8) and with more time to homoplasmy (at passage 10). However, another sibling, also with U5a, generated by the same maternal and donor mtDNA combination did not show a reversal [4], suggesting that other unknown factors beyond the haplogroup are critical.

Getting back to our recent case, the mtDNA haplogroup of the patient and the donor oocyte were I and L2c, respectively. The mother was a carrier for the T8993G mutation. Previous studies demonstrated that the heteroplasmy level for this particular mutation (T8993G) remains stable during pregnancy [11]. The CSBII SNP sequences were G6AG7 in both the mother and the donor. Considering the possibility of mtDNA heteroplasmy drift during cell proliferation and differentiation in the boy, we examined the heteroplasmy level of T8993G in all the possible tissues which could be obtained without performing invasive procedures. The mutation load for T8993G varied slightly in these tissues from undetectable in the placenta, umbilical blood and umbilical cord, to 2.36% in the urine precipitate, 3.52% in the buccal epithelium, 5.59% in the hair follicles, 6.77% in the amnion and 9.23% in the circumcised foreskin. Although these results suggest that the maternal mtDNA transmission rate varies slightly among tissues, in general, six out of eight tissue samples we tested showed less mtDNA T8993G mutation load than 5.73% or fluctuated around the 5.73% found in the trophectoderm biopsy of the blastocyst embryo. There is no evidence to support that maternal mutated mtDNA had a selective advantage during embryo development in this case. Furthermore, for this particular heteroplasmic T8993G mutation, the genotype-phenotype correlation has been well studied in a very large cohort [16]. The data from 48 T8993G mutation pedigrees and 178 individuals showed that the probability of having severe symptoms is very low until the mutation load reaches 60%–70%. It has also been reported for this particular mutation that there is no substantial tissue variation and no increase in heteroplasmy level over time [18]. In our study, the mother’s whole mtDNA sequencing analysis revealed T8993G heteroplasmy levels of 23.27%, 24.50%, and 33.65% in her hair follicles, blood, and urine precipitate, respectively and she remains asymptomatic. Hence, with this heteroplasmy level (<10%), there is considerable optimism that medical problems will not arise due to the mtDNA mutation, and indeed, the baby is currently healthy at 11 months of age.

## Going Forward

Although there is little concern regarding mtDNA heteroplasmy drift due to the novelty of the procedure, we will follow the child closely in the coming years. Our quite extensive plan will provide a degree of assurance that helped to clear the final regulatory hurdle in the United Kingdom in the approval of mitochondrial replacement therapy [26]. Our current protocol recommends that the baby is to be followed by a pediatrician and medical genetics specialist, in addition to the routine pediatrics follow-up. The following is the detailed medical plan schedule:

1. In the first year, the child will be followed every 3 months.
2. In the second year, the child will be followed every 6 months.
3. From the third year on, the child will be followed annually until 18 years old if the child is asymptomatic.
4. After 18 years old, fertility function will be followed.
5. The child’s parent or legal guardian will fill a questionnaire every three months, which is attached, specific for mitochondria disease.
6. If the child presents with any symptom, he will be referred to a pediatrician and medical genetics specialist in our research team for further evaluation at the University of Cincinnati Children’s Hospital.
7. Besides routine physical exams, blood tests, and radiology studies, the parents or guardians of the child consent to do tests which are specific for mitochondria-related diseases. The tests include, but are not limited to, hearing tests, MRI (if there is a need), muscle biopsy and electromyography.

Obviously, the heteroplasmic T8993G mutation is our primary concern in this situation. However, we are optimistic that this mutation will remain a non-issue going forward, as genotype-phenotype correlation studies have shown that the heteroplasmy levels of the T8993G mutation do not vary significantly and remain constant over time [16,18]. As for potential differences in the nuclear genome of the patient and donor, for the purposes of this case, we did not perform sequencing of the nDNA of either individual, as there are millions of SNP variants between the nDNA of any two individuals. Although this may become a standard practice in the future as genomic technologies continue to mature, such an effort would have been largely fruitless in this case without a focused hypothesis to narrow down which variants to investigate. In contrast, the entire mitochondrial genome sequence was easily obtained and analyzed, and its importance for this specific procedure is unquestionable.

In summary, we have attempted to use this review as a way of reflecting on issues that may deserve more consideration with respect to utilizing oocyte spindle transfer or nuclear transfer procedures in the future. One thing that may bear particular consideration is the issue of haplogroup matching between the patient/mother and the oocyte donor, which were not closely matched in our case. Part of the reason for this situation is a lack of strong scientific evidence that haplogroup matching would be of any major benefit. Haplogroup matching was definitely not at the forefront of debate at the time of the procedure as it is now. Of course, haplogroup matching is unlikely to produce any harm. However, we must balance the match between the patient and donor haplogroups against other factors, such as the limited availability of donor eggs and the existence of so-called “private” mtDNA variants that differ even within haplogroups. All of these considerations must be accounted for when matching patient and donor, and in situations where they conflict, a judgment call must be made as to which factors are most critical. For instance, if the choice was between matching the haplogroup or matching the CSBII sequence (which can vary within a haplogroup), we would strongly argue that matching the latter takes precedence over the former, for the reasons stated above. Additional research will, of course, be crucial in resolving these matters. Most importantly, when making these evaluations, one must exercise the utmost care when using ES studies to determine clinical decisions in terms of fertility and human development, as mitochondrial behavior is often significantly different in embryonic stem cells compared to normal human development [5].
